# Obtaining structured clinical data from unstructured data using natural language processing software

**DOI:** 10.23889/ijpds.v1i1.381

**Published:** 2017-04-19

**Authors:** Arron S Lacey, Beata Fonferko-Shadrach, Ronan A Lyons, Mike P Kerr, David V Ford, Mark I Rees, Owen W Pickrell

**Affiliations:** 1 Swansea University; 2 Cardiff University

## Background

Free text documents in healthcare settings contain a wealth of information not captured in electronic healthcare records (EHRs). Epilepsy clinic letters are an example of an unstructured data source containing a large amount of intricate disease information. Extracting meaningful and contextually correct clinical information from free text sources, to enhance EHRs, remains a significant challenge. SCANR (Swansea University Collaborative in the Analysis of NLP Research) was set up to use natural language processing (NLP) technology to extract structured data from unstructured sources.

IBM Watson Content Analytics software (ICA) uses NLP technology. It enables users to define annotations based on dictionaries and language characteristics to create parsing rules that highlight relevant items. These include clinical details such as symptoms and diagnoses, medication and test results, as well as personal identifiers.

## Approach

To use ICA to build a pipeline to accurately extract detailed epilepsy information from clinic letters.

## Methods

We used ICA to retrieve important epilepsy information from 41 pseudo-anonymized unstructured epilepsy clinic letters. The 41 letters consisted of 13 `new' and 28 `follow-up' letters (for 15 different patients) written by 12 different doctors in different styles. We designed dictionaries and annotators to enable ICA to extract epilepsy type (focal, generalized or unclassified), epilepsy cause, age of onset, investigation results (EEG, CT and MRI), medication, and clinic date. Epilepsy clinicians assessed the accuracy of the pipeline.

## Results

The accuracy (sensitivity, specificity) of each concept was: epilepsy diagnosis 98% (97%, 100%), focal epilepsy 100%, generalized epilepsy 98% (93%, 100%), medication 95% (93%, 100%), age of onset 100% and clinic date 95% (95%, 100%).

Precision and recall for each concept were respectively, 98% and 97% for epilepsy diagnosis, 100% each for focal epilepsy, 100% and 93% for generalized epilepsy, 100% each for age of onset, 100% and 93% for medication, 100% and 96% for EEG results, 100% and 83% for MRI scan results, and 100% and 95% for clinic date.

## Conclusions

ICA is capable of extracting detailed, structured epilepsy information from unstructured clinic letters to a high degree of accuracy. This data can be used to populate relational databases and be linked to EHRs. Researchers can build in custom rules to identify concepts of interest from letters and produce structured information. We plan to extend our work to hundreds and then thousands of clinic letters, to provide phenotypically rich epilepsy data to link with other anonymised, routinely collected data.

